# At the end of the mandate

**DOI:** 10.22336/rjo.2021.64

**Published:** 2021

**Authors:** Mircea Vasile Filip

**Affiliations:** * Assoc. Prof., MD. RSO President 2012-2021

For the last 9 years I have been the President of the Romanian Society of Ophthalmology, meaning 2 mandates and a little bit more, due to the pandemics.

A long period of time, but a fulfilling one, from my point of view!

The decisions were taken easier due to the collaboration, the permanent exchange of ideas, the fact that we know and we appreciate each other.

Annual congresses with significant participation, reunited with SEEOS (South-East European Society of Ophthalmology) and/ or BSOS (Black Sea Ophthalmological Society), with well-known national and international speakers were organized. The conferences in Sinaia were very much appreciated by the colleagues and now we all hope to meet again there in 2022 and not on Zoom.

Beside the paper works presented at the Romanian Society of Ophthalmology (RSO) Congresses, prize awarding contests for residents and nurses were organized. Previously, the participations were honorific but we changed this and offered significant money prizes and the results were seen. On the other hand, some companies offered scholarships in different European clinics and this was very much appreciated by the young doctors.

The Society bought the educational “One network” programme from the American Academy of Ophthalmology for professional purposes, which was well received by the Romanian ophthalmologists.

The Society was preoccupied by the national organization of the International Council of Ophthalmology exams, with significant reduced taxes because they were collected by RSO.

A major decision with significant impact in our collectivity and international appreciation was the transformation of “Oftalmologia” journal into “Romanian Journal of Ophthalmology”, published in English. One of the results of this change was the fact that we received for publishing many articles from different parts of the world.

The implementation of a new curricula for specialist doctor exam, requested by the University staff for the past few years, was obtained with great effort and in collaboration with the Ministry of Health.

A new headquarter situated in the central part of Bucharest was bought for the Society, facilitating the collaboration with the doctors, medical companies, and authorities.

It was a constant and fulfilling collaboration with the medical companies, companies that were involved financially. Without them, the Society’s activities would have been poorer. Their help made possible a series of online webinars, organized at high standards and their activity continues in the present and hopefully will also continue in the future.

In conclusion, it must be said that the good, collegial, and responsible collaboration was a constant in the activity of RSO board and now we confidently look to the future leadership.


**With friendship and consideration,**
**Assoc. Prof. Mircea Vasile Filip, MD**
**RSO President 2012-2021**


